# Cervical precancer screening with HPV DNA testing and mobile colposcopy in women with sickle cell disease in Accra, Ghana

**DOI:** 10.3332/ecancer.2023.1571

**Published:** 2023-07-12

**Authors:** Yvonne Dei-Adomakoh, Kofi Effah, Ethel Tekpor, Selina Crabbe, Joseph Emmanuel Amuah, Comfort Mawusi Wormenor, Georgina Tay, Eugenia Vicky Asare, Seyram Kemawor, Stephen Danyo, Esu Aku Catherine Morkli, Faustina Tibu, Nana Owusu Essel, Patrick Kafui Akakpo

**Affiliations:** 1 Korle-Bu Teaching Hospital, Ghana Institute of Clinical Genetics, Accra, Ghana; 2 Department of Haematology, University of Ghana Medical School, Accra, Ghana; 3 Catholic Hospital, Battor, Ghana; 4 Faculty of Medicine, School of Epidemiology and Public Health, University of Ottawa, Ottawa, ON K1H 8M5, Canada; 5 Department of Haematology, Korle-Bu Teaching Hospital, Accra, Ghana; 6 Department of Emergency Medicine, Faculty of Medicine & Dentistry, College of Health Sciences, University of Alberta, Edmonton, AB T6G 2T4, Canada; 7 Department of Pathology, Clinical Teaching Center, School of Medical Sciences, University of Cape Coast, Cape Coast, Ghana; a https://orcid.org/0000-0002-2017-2569; b https://orcid.org/0000-0003-1216-2296; c https://orcid.org/0000-0001-5494-5411; d https://orcid.org/0000-0003-0356-0663

**Keywords:** sickle cell disease, human papillomavirus infection, uterine cervical neoplasm, haemoglobinopathy, colposcopy, human papillomavirus DNA test

## Abstract

**Background:**

Worldwide, about 20–25 million people are affected by sickle cell disease (SCD), with 60% of patients living in sub-Saharan Africa. Despite recent therapeutic advancements resulting in improved life expectancy among SCD patients, the prevalence of high-risk human papillomavirus (hr-HPV) and cervical lesions have not been studied in women with SCD. We determined the prevalence of hr-HPV and cervical lesions among women with SCD and recommended strategies for reducing cervical cancer incidence in this cohort.

**Methods:**

Through the mPharma 10,000 Women Initiative, women with SCD attending routine visits at the Ghana Institute of Clinical Genetics SCD clinic were screened by trained nurses. Screening was performed via concurrent MA-6000 hr-HPV DNA testing and enhanced visual assessment (EVA) mobile colposcopy from mobileODT.

**Results:**

Among 168 participants screened (mean age, 43.0 years), the overall prevalence rates of hr-HPV infection and cervical lesions were 28.6% (95% CI, 21.7–35.4) and 3.6% (95% CI, 0.8–6.4), respectively. The hr-HPV prevalence rates stratified by haemoglobin genotype were 29.4% (95% CI, 19.7–39.1) and 28.6% (95% CI, 18.5–38.7) for genotypes SS and SC, respectively. None of the five women with the SF genotype tested hr-HPV positive, and the only patient with Sbthal genotype tested hr-HPV positive. Two women were EVA ‘positive’ but hr-HPV negative, whereas four were EVA positive and hr-HPV positive. Exploratory analysis revealed no significant associations between hr-HPV positivity and age, education level, marital status or parity.

**Conclusion:**

In the absence of a comprehensive national cervical screening programme aimed at including women with SCD as a special population, cervical cancer may increase in frequency among SCD patients. Thus, there is a need to build capacity and expand the scope of screening services for women with SCD.

## Introduction

Sickle cell disease (SCD) is a common haemoglobinopathy that results from a genetic mutation in the β-globin gene. SCD occurs in 1.8% of Ghana-born newborns, and approximately 300,000 babies are born each year in sub-Saharan Africa [1]. Infection is a major complication of SCD, causing significant morbidity and mortality in Africa [2–5]. In the past, SCD was considered a childhood disease because few affected adolescents survived to adulthood [6]. However, its epidemiology has largely evolved over the last few decades, with many patients surviving into their fifth decade [6, 7]. Reasons for this improvement in survival include widespread universal newborn screening, parental education about expectant management of fever and sequestration crises, vaccination against *Streptococcus pneumoniae* and type b *Haemophilus influenzae* and hydroxyurea administration [7, 8]. As patient survival has improved over the years, there are concerns about diseases typically associated with maturity among them. Specifically, SCD is strongly linked to infection, chronic inflammation and reduced immunity, which may predispose them to several cancers [9–12]. Studies have found an increased incidence of certain haematological and solid malignancies among SCD patients compared to general or hospitalised populations [13, 14].

In SCD, sustained red blood cell sickling may result in a vicious cycle of impaired blood flow, hypoxia, premature breakdown of red blood cells with iron release and serious sequelae of irreversible damage to end organs with severe long-term consequences [15]. Chronic haemolysis disrupts the iron balance and causes oxidative stress and inflammation, damaging proper immune functioning [16]. These point to defects in adaptive immunity, increased haematopoietic cell turnover, chronic inflammation and reduced immune function, leading researchers to speculate that cancer risk may be higher in this group [13]. Chronic antigenic stimulation through recurrent infections is a predisposing factor for cancer [17]. In addition, hypoxia and high interstitial fluid pressure have been shown to independently predict nodal and distant metastases and survival in patients with cervical cancer [18].

In Ghana, an estimated 2,797 women are diagnosed with cervical cancer annually, and more than 1,699 women die from the disease yearly [19]. Despite this, there is an absence of population-based studies on cervical precancer risk among women with SCD [20]. The question also remains if SCD patients have an increased incidence of high-risk human papillomavirus (hr-HPV) infection and, thus, cervical precancerous lesions.

To answer these questions, we determined the prevalence of hr-HPV infection and cervical lesions in a cohort of women who were enrolled in an SCD clinic in Accra, Ghana, and underwent cervical precancer screening. Specifically, we compared their estimated prevalence to the general female population of Ghana, stratified the prevalence according to sickle cell genotype, and discussed potential strategies for targeting this cohort.

## Materials and methods

### Study design

We conducted the present retrospective descriptive cross-sectional study to investigate the prevalence of hr-HPV infection and cervical lesions among women living with SCD who underwent cervical screening by way of concurrent hr-HPV testing and visual inspection using the EVA mobile colposcope from MobileODT, Tel Aviv, Israel, in an outpatient setting in Accra, Ghana. The screening exercises were undertaken as part of the mPharma company’s 10,000 women campaign to screen 10,000 women in Ghana and Nigeria using HPV DNA testing.

### Study setting and participants

This study involved the screening of 168 women with SCD aged ≥20 years who volunteered to be registered and screened during March–May 2022. These women were regular attendees of the SCD clinic held at the Ghana Institute of Clinical Genetics (GICG), Korle-Bu Teaching Hospital, Accra, Ghana. The GICG provides care to patients with SCD on an outpatient basis. As the premier adult SCD clinic in the country, the GICG SCD clinic sees patients from all over Ghana, with a large majority coming from Southern Ghana. As of 2022, the clinic had registered more than 25,000 adolescents and adults with SCD [21]. The clinic sees 10,000–15,000 patients annually and a mean of 50 patients per day [21]. Patients needing additional specialist care or in-patient services are referred from the GICG clinic to the Korle-Bu Teaching Hospital.

### Ethical considerations

Ethical clearance was given by the Scientific and Technical Committee/Institutional Review Board of the Korle-Bu Teaching Hospital (approval no. KBTH-STC/IRB/000175/2022). All study participants provided verbal consent before questionnaire administration, cervical screening and sample collection.

### Sample size

We did not calculate an optimum sample size before initiating the study primarily because the screening exercise and procedures were not initially performed in the context of a research study but as service provision. Further, we did not find any epidemiological studies of cervical cancer risk in women with SCD; thus, no baseline data could objectively guide such a calculation. Instead, we included all women attending the SCD clinic who were eligible, willing and able to consent to screening.

### Data collection and outcomes

Each woman was provided with detailed information on the need for cervical screening, as well as the procedures to be performed and their associated benefits and risks. After giving verbal informed consent to participate, a nurse collected data on sociodemographic and clinical information using a structured questionnaire routinely used at the Cervical Cancer Prevention and Training Centre (CCPTC), Battor, Ghana. Then, cervical screening was performed via cervical sampling for hr-HPV DNA testing and EVA mobile colposcopy during the same visit. Cervical specimens were then submitted to the central laboratory of the CCPTC for testing with the MA-6000 hr-HPV DNA platform (Sansure Biotech Inc., Hunan, China) within 7 days after sample collection. Data collected using the questionnaire and screening outcomes were entered into REDCap version 11.0.3 (Vanderbilt University, Nashville, TN, USA) and stored in secure databases managed by the CCPTC. The databases were then queried, and data were extracted and anonymised before the statistical analyses. The outcome of interest in this study was the presence of a clinically-relevant lesion(s) on EVA mobile colposcopy or a positive hr-HPV DNA test.

### Cervical HPV specimen collection, EVA colposcopy and treatment

Well-trained and experienced nurses from the CCPTC performed cervical sampling and mobile colposcopy professionally. After placing the woman in the dorsal lithotomy position, the nurse inserted a sterile vaginal speculum to expose the cervix and used a cytobrush to take the cervical specimen in gentle motion. The sample was then placed in a specimen collection tube, capped and sent for laboratory processing and typing. The nurse performed a colposcopy within the same screening session using the EVA device. The results of EVA colposcopy were interpreted as ‘positive’ in the presence of a clinically relevant lesion or ‘negative’ otherwise. Women with cervical lesions were managed either conservatively if they had minor changes or offered the option of thermal ablation onsite. Women with lesions not amenable to ablation were offered a loop electrosurgical excision procedure (LEEP) performed by a specialist gynaecologist. A prior publication has described the algorithms used for cervical screening and triaging with EVA mobile colposcopy [22].

### Laboratory testing: MA-6000 HPV DNA extraction and PCR assay

Cervical specimens obtained for MA-6000 testing were processed per the manufacturer’s instructions [23] and as detailed elsewhere [24]. In brief, following the isolation of a pure fraction of DNA in solution, a sample release reagent was added, mixed and incubated at room temperature for 10 minutes. DNA amplification was performed after a series of 45 polymerase chain reaction (PCR) cycles. Fluorescence data were collected during the amplification step. The qualitative MA-6000 kit is configured to detect four dyes: the FAM dye (for HPV 18), CY5 dye (for HPV 16), HEX dye (which detects beta-globin as an internal control) and ROX dye (for the collective determination of HPV 31/33/35/39/45/51/52/53/56/58/59/66/68 as hr-HPV DNA). The test results were read and interpreted according to the manufacturer’s guidelines.

### Statistical analysis

We used frequencies and percentages to summarise the study participants' categorical sociodemographic (e.g., education level, income status and marital status) and clinical (e.g., hr-HPV positivity, clinical history and findings on cervical inspection) variables. Symmetrically-distributed continuous variables are presented as means and standard deviations (SDs), whereas skewed variables are summarised using medians and interquartile ranges (IQRs). Box plots describe the age distribution of women with SCD by hr-HPV positivity status. The chi-squared test of independence was used to explore the relationship between hr-HPV positivity and selected sociodemographic factors. The overall prevalence rate of hr-HPV infection and prevalence rates stratified by sickle cell genotype with their 95% confidence intervals (CIs) are presented as percentages. Due to the low number of women with cervical lesions, the exact binomial 95% CI is presented for the overall prevalence of cervical lesions without stratification. All statistical analyses were performed using Stata 14.2 (StataCorp LLC, College Station, TX, USA). Hypothesis tests were performed at a two-sided significance level of 5%.

## Results

### Patient recruitment and selection

**Figure 1.**
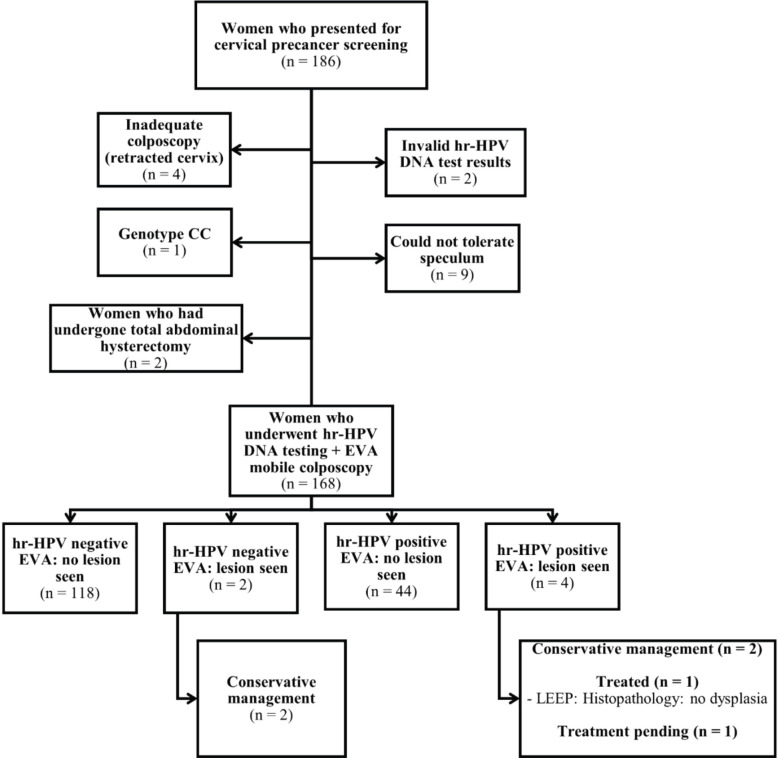
Flow chart for cervical precancer screening among women attending the sickle cell clinic. hr-HPV, high-risk human papillomavirus; LEEP, loop electrosurgical excision procedure; CIN, cervical intraepithelial neoplasia.

A flow chart of patient recruitment and screening is shown in Figure 1. A total of 186 women attending the SCD clinic consented to undergo cervical screening. Of these, four could not be screened using both hr-HPV DNA testing and EVA colposcopy due to retracted cervix; one had CC genotype, two had invalid hr-HPV DNA test results (each sample was tested twice, and both led to weak results), nine could not tolerate a small speculum despite adequate lubrication and two had undergone total abdominal hysterectomy. Thus, the remaining 168 patients underwent cervical screening via concurrent hr-HPV DNA testing and EVA mobile colposcopy. Among the 168 SCD patients, the commonest genotype was SS (*n* = 85, 50.6%); 77 women (45.8%) had SC genotype, 5 (3.0%) had SF genotype and 1 (0.6%) had Sbthal genotype.

### Sociodemographic and clinical characteristics of the study participants

The average age of the women was 43.0 years (95% CI, 41.0–45.0) with a median parity of 1 (IQR: 0, 2). Forty percent of the women were married, 35.1% were single, 10.1% were widows, 8.9% had a steady partner and 6.0% were divorced. A majority of participants (60.7%) had at least a secondary level education, and most (78.6%) earned an income (Table 1). A minority (16.1%) of the women were currently using some form of contraceptive, and 35.7% had used contraception. Most (87.5%) were Christians, and the remaining were Muslims. None of the women reported smoking or drinking alcohol. A majority (58.9%) of them did not know their Human Immunodeficiency Virus (HIV) status, and the remaining 41.1% reported a negative HIV status. A small minority of patients had a history of hypertension (9.5%), diabetes mellitus (1.8%) or asthma (1.2%). A small group (6.0%) had undergone prior cervical screening. All 168 women showed normal findings on vulval and vaginal examination, and 3 (1.8%) showed abnormal on a gross (non-colposcopic) cervical inspection. One woman with a ‘blue lesion’ on the cervix was sent to see a gynaecologist for a possible biopsy. Two women had cervical polyps.

### Overall screening outcomes and treatment of study participants

The hr-HPV positivity rate among the 168 SCD patients screened via concurrent hr-HPV DNA testing and EVA mobile colposcopy was 28.6% (95% CI, 21.7–35.4). When stratified by sickle cell genotype, the hr-HPV prevalence rates were 28.6% (95% CI, 18.5–38.7) among women with SC genotype and 29.4% (95% CI, 19.7–39.1) among those with SS genotype (Table 2). None of the five women with the SF genotype (0.0%) tested hr-HPV positive, and the only patient with Sbthal genotype tested positive for hr-HPV.

On EVA colposcopy, six women showed clinically relevant lesions; thus, the overall prevalence of cervical lesions was 3.6% (95% CI, 0.8–6.4). Two women were EVA ‘positive’ but hr-HPV negative, whereas four were EVA ‘positive’ and hr-HPV positive. As of the time of this report, one of the six women with cervical lesions in the latter group had been treated using LEEP, four were managed conservatively and one had treatment pending despite being contacted multiple times to come for treatment.

### Exploratory analysis of factors associated with hr-HPV positivity among women with SCD

**Figure 2.**
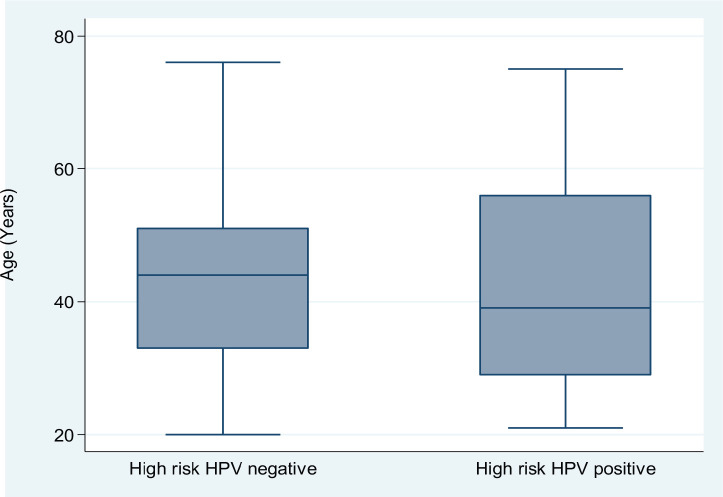
A side-by-side box and whisker plot showing the age distribution of patients attending a sickle cell clinic by high-risk HPV positivity status.

Next, we explored the association between hr-HPV positivity and selected sociodemographic characteristics among the study participants. hr-HPV-positive SCD patients tended to be slightly younger than their hr-HPV-negative counterparts (Figure 2); however, there was no significant difference in age between the two subgroups (41.4 (SD, 15.1) versus 43.7 (SD, 12.3) years; Student *t*-test *p* = 0.3064)). Compared to married women with SCD, unmarried women were more likely to test hr-HPV positive (odds ratio (OR) = 1.58; 95% CI, 0.75–3.43; *p* = 0.1973). Further, relative to women with an education level below the secondary school, those with at least a secondary school education showed an OR of 1.43 (95% CI, 0.67–3.1;* p* = 0.3177) for hr-HPV positivity. On the other hand, compared to women with a parity of ≤1, those with a parity of ≥2 showed an OR of 0.78 (95% CI, 0.37–1.61* p* = 0.4639) for hr-HPV positivity. Thus, in this cohort of SCD patients, hr-HPV positivity was not significantly associated with age, education level, marital status or parity.

## Discussion

This study aimed to estimate the prevalence of hr-HPV infection and cervical lesions among women with SCD who underwent cervical screening via concurrent hr-HPV DNA testing and EVA mobile colposcopy at an outpatient SCD clinic in Ghana. To the best of our knowledge, no prior research has been conducted in such a cohort or attempted to assess the risk of cervical precancer and cancer among women with SCD. Our study draws on the need to document the epidemiology and risk of cervical cancer in patients with SCD, given that many survive into adulthood due to therapeutic advancements that have improved their life expectancy. Here, we found an overall hr-HPV prevalence of 28.6% with only minor variations among sickle cell genotypes (28.6% and 29.4% for SC and SS, respectively). In addition, 2.4% tested both hr-HPV and EVA ‘positive’, while EVA colposcopy revealed clinically relevant cervical lesions in 3.6%. Exploratory analysis revealed no significant associations between hr-HPV positivity and age, level of education, marital status or parity.

Although we could not identify any prior screened SCD cohorts to compare our findings, the overall and haemoglobin genotype-stratified hr-HPV prevalence rates among our study participants were higher than the expected prevalence of 21.3% reported by the World Health Organization (WHO) for West African women [25, 26]. Statistics about hr-HPV prevalence in Ghana vary widely, with limited comparability due to differences in specimen collection methods and testing platforms (with varying sensitivities and genotyping). In addition, HPV prevalence estimates may differ considerably based on the approach to participant selection (e.g., randomised sampling versus opportunistic selection from clinical settings, as used in our study) [27]. As it stands, the overall and sickle cell genotype-stratified hr-HPV prevalence rates in our study were lower than those reported among community-dwelling women with a similar age distribution in the North Tongu District of Ghana (32.3%; 95% CI, 30.2–34.5) [25] and women attending a reproductive health clinic in Ghana (35.0%; 95% CI, 29.6–40.4) [28]. It is worth mentioning that some differences in platform genotyping potentially limit these comparisons. Specifically, the MA-6000 platform used in this study does not distinguish among recognised (HPV 16/18/31/33/35/39/45/51/52/56/58/59), potential (HPV 53) and probable (HPV 66/68) hr-HPV genotypes [24]. On the other hand, and in support of the comparison to the WHO estimate, the prevalence rates identified exceeded the 19.7% aggregate positivity rate (95% CI, 17.2–22.4) reported in a study conducted among community-dwelling women in Ibadan, Nigeria, in which probable and potential hr-HPV genotypes were combined with recognised genotypes [29].

In addition to the elevated cervical precancer risk (judged solely based on hr-HPV estimates in this study), a number of physiologic abnormality characteristics of SCD could potentially worsen the prognoses of cervical precancerous and cancerous lesions among SCD patients. First, erythrocyte deformability may reduce in the presence of acidosis, as frequently observed in cervical cancer and other solid malignancies [30, 31]. Hypoxia in precancerous and cancerous lesions among SCD patients also significantly lowers their disease-free survival compared to tumours with normal oxygenation levels, regardless of size and location [30]. Hypoxia is also an essential determinant of prognosis in cervical cancer that influences patient survival following radiotherapy or surgical management [32]. Further, it induces changes in tumour gene expression, resulting in more aggressive tumour phenotypes with worse potential for metastasis [30].

Apart from the possible changes in the precancer tumour microenvironment mediated by chronic inflammation and oxidative stress [16], these differences in physiology compared to women with normal haemoglobin genotypes make a case for the development of strategies mainly aimed at including women with SCD in cervical precancer screening programmes as a special population. Nine (4.9%) of the initial 185 women (women with CC genotype were excluded as CC without S genotype is not classified as SCD) could not be screened because they could not tolerate a small-sized speculum despite lubrication. This might be related to the findings of a recent study that chronic pain in premenopausal women with SCD is associated with dyspareunia [33]. While this observation’s mechanism is multifactorial, it may be mediated by chronic inflammation, nerve damage, long-term opioid use and persistent painful input, which result in neuroplastic changes and sensitisation to pain [34]. The medical literature describes gnathopathy, frontal and parietal bossing, finger clubbing, long extremities and spider fingers as associated with SCD [35–39]. No literature on general vaginal or cervical features in women with SCD exists. The impression of the screening team was that many of the women with SCD had pale, dry vaginas and small cervices. Many had arthritis of the hip joints (possibly from avascular necrosis of the femoral heads), making it difficult to put them in the lithotomy position for screening. This will be an area to look into in future extensive studies in women with SCD. Another challenge in a screening programme involving women with SCD pertains to managing screen positives due to low haemoglobin levels. Bleeding during treatment and infection after treatment could lead to severe morbidity. For instance, treatment via LEEP for one woman in our study who had a cervical lesion was delayed because she persistently had a haemoglobin level of less than 8 g/dL, and we feared that a bit of bleeding during and after LEEP would lead to severe complications, including haemorrhagic shock. With the current lack of high-quality evidence, we cannot determine the magnitude and scope of poor outcomes or optimal clinical strategies for managing cervical lesions among women with SCD genotypes, particularly those presenting with persistent anaemia.

## Limitations

Our study had some limitations. First, the participants were drawn from a convenience sample of women who presented for routine visits at the clinic. Thus, they may not represent the entire clinic population or all women with SCD in Ghana. Second, we did not differentiate between transient and persistent hr-HPV infections among the participants. Third, we did not perform full genotyping for hr-HPV-positive women due to logistic and resource limitations constraints, thereby preventing complete hierarchical analysis of genotypes according to their known associations with cervical cancer. Further, due to the small sample size and having performed the screening exercise outside a research setting, we could not screen adequate numbers of women with SF and Sbthal genotypes to estimate hr-HPV prevalence rates among them accurately.

This work was part of the mPharma 10,000 Women Initiative which was to give 10,000 women in Ghana and Nigeria free HPV DNA testing. Unfortunately, we were not given enough funds for HIV testing and biopsies of all lesions, so the plan was to follow up with women with minor changes (thin acetowhitening) suggestive of CIN 1 in 6–12 months unless there was a high risk of loss to follow up. This was to prevent overtreatment. Women with major changes (dense acetowhitening) suggestive of CIN II/III were treated.

## Conclusion

Due to therapeutic advancements in the care of SCD patients, increasing their life expectancy, and in the absence of a comprehensive national cervical screening programme that aims to include women with SCD as a special population, we posit that cervical cancer may increase in incidence among SCD patients. Thus, there is a need to build capacity and expand the scope of screening services offered for women with SCD.

## Conflicts of interest

The authors declare that they have no conflict of interest.

## Funding

This work was part of the mPharma 10,000 Women Initiative which was to give 10,000 women in Ghana and Nigeria free HPV DNA testing. None of the authors has any financial interests to declare.

## Author contributions

Conceptualization and study design: KE, ET, CMW, YDA, and JEA. Screening and data collection: ET, CMW, GT, FT, SC, EACM, SK, SD, and KE. Data management and formal analysis: JEA, SD, CMW, ET, NOE, and KE. Writing – original draft: NOE, YDA, JEA, ET, KE, PKA, and EVA. All the authors read and approved the manuscript in its current form.

## Notation of prior abstract presentation

This work has been partially presented as an abstract poster at the 35th International Papillomavirus Conference in Washington, DC, from 17 to 21 April 2023.

## Figures and Tables

**Table 1.** Sociodemographic and clinical characteristics of women attending an SCD clinic who underwent cervical screening (*n* = 168).

Sociodemographic variables	Estimate
Age, mean (SD)	43.0 (13.1)
Parity, median (IQR)	1 (0, 2)
Religion, *n* (%)	
Christian	147 (87.5)
Muslim	21 (12.5)
Marital status, *n* (%)	
Divorced	10 (6.0)
Married/cohabiting	67 (39.9)
Single	59 (35.1)
Widowed	17 (10.1)
Has a steady partner	15 (8.9)
Education level, *n* (%)	
None	9 (5.4)
Primary	53 (31.6)
Secondary	38 (22.6)
Tertiary	64 (38.1)
Vocational/commercial/other	4 (2.4)
Earns an income, *n* (%)	132 (78.6)
Monthly income, GH¢; *n* (%)	
<100	22 (13.1)
100–250	24 (14.3)
250–500	13 (7.7)
>500	53 (31.6)
No income/prefer not to say	56 (33.3)
The primary mode of medical bill payment, *n* (%)	
NHIS	121 (71.6)
Relative/out-of-pocket/other	48 (28.4)
Alcohol intake, *n* (%)	0 (0.0)
Ever smoked, *n* (%)	0 (0.0)
Past contraceptive use, *n* (%)	60 (35.7)
Current contraceptive use, *n* (%)	27 (16.1)
Clinical/surgical characteristics	
HIV status, *n* (%)	
Negative	69 (41.1)
Unknown	99 (58.9)
Past medical history	
Hypertension, *n* (%)	16 (9.5)
Diabetes mellitus, *n* (%)	3 (1.8)
Asthma, n (%)	2 (1.2)
Tuberculosis, *n* (%)	0 (0.0)
Previous HPV vaccination, *n* (%)	0 (0.0)
Vulval inspection findings, *n* (%)	
Normal	168 (100.0)
Abnormal	0 (0.0)
Vaginal examination, *n* (%)	
Normal	168 (100.0)
Abnormal	0 (0.0)
Previous cervical screening, *n* (%)	10 (6.0)
Previous cervical treatment, *n* (%)	0 (0.0)
Cervical inspection, *n* (%)	
Normal	165 (98.2)
Abnormal	3 (1.8)
Adequate for EVA mobile colposcopy, *n* (%)	168 (100)

SCD, sickle cell disease; SD, standard deviation; IQR, interquartile range; NHIS, National Health Insurance Scheme; HIV, human immunodeficiency virus; HPV, human papillomavirus

**Table 2.** Prevalence of hr-HPV infection and cervical lesions among SCD patients who underwent cervical precancer screening via concurrent hr-HPV DNA testing and EVA mobile colposcopy (*n* = 168).

Clinical outcome variables	Prevalence (%)	95% CI
hr-HPV+ (all patients)	28.6	21.7–35.4
hr-HPV+ (patients with SS genotype)	29.4	19.7–39.1
hr-HPV+ (patients with SC genotype)	28.6	18.5–38.7
hr-HPV+ (patients with SF genotype)	0.0	-
Cervical lesions	3.6	0.8–6.4

hr-HPV, high-risk human papillomavirus; SCD, sickle cell disease; CI, confidence interval
